# Arterial spin labeling for moyamoya angiopathy: A preoperative and postoperative evaluation method

**DOI:** 10.1515/tnsci-2022-0288

**Published:** 2023-06-07

**Authors:** Sun Yuxue, Wang Yan, Xue Bingqian, Liang Hao, Li Chaoyue

**Affiliations:** Department of Neurosurgery, Henan Provincial People’s Hospital (Zhengzhou University People’s Hospital, Henan University People’s Hospital), Zhengzhou, China; Department of Radiology, Henan Provincial People’s Hospital (Zhengzhou University People’s Hospital, Henan University People’s Hospital), Zhengzhou, China; Department of Neurosurgery, Henan University People’s Hospital (Henan Provincial People’s Hospital), Zhengzhou, China

**Keywords:** arterial spin labeling technology, cerebral blood flow, moyamoya angiopathy, perioperative period, prognosis

## Abstract

**Objectives:**

Studies have shown that arterial spin labeling (ASL) effectively replaces traditional MRI perfusion imaging for detecting cerebral blood flow (CBF) in patients with Moyamoya angiopathy (MMA). However, there are few reports on the relationship between neovascularization and cerebral perfusion in patients with MMA. The aim of this study is to investigate the effects of neovascularization on cerebral perfusion with MMA after bypass surgery.

**Methods:**

We selected patients with MMA in the Department of Neurosurgery between September 2019 and August 2021 and enrolled them based on the inclusion and exclusion criteria. ASL imaging was used to monitor the baseline CBF level before surgery and determine the changes in cerebral vessels at postoperative 1 week and 6 months, respectively. The Alberta stroke grade, modified Rankin Scale (mRS), and digital subtraction angiography images were used to evaluate the effect of postoperative CBF status and prognosis. Ninety hemispheres from 51 patients were included in this study. There were no significant differences in the baseline data of the enrolled patients. At 1 week and 6 months post-surgery, the CBF state in the operation area was significantly changed compared with that at baseline (*P* < 0.05). The preoperative Alberta score (*t* = 2.714, *P* = 0.013) and preoperative mRS score (*t* = 6.678, *P* < 0.001) correlated with postoperative neovascularization.

**Conclusion:**

ASL is an effective method for detecting CBF and plays an important role in the long-term follow-up of patients with MMA. Combined cerebral revascularization significantly improves CBF in the operation area both in the short and long terms. Patients with lower preoperative Alberta scores and higher mRS scores were more likely to benefit from combined cerebral revascularization surgery. However, regardless of the type of patient, CBF reconstruction can effectively improve prognosis.

## Abbreviations

ASL: arterial spin labeling
ASPECT: Alberta Stroke Program Early CT Score
CBF: cerebral blood flow
CHD: chronic heart disease
DSA: digital subtraction angiography
MCA: middle cerebral artery
MMA: Moyamoya angiopathy
MR: magnetic resonance
mRS: modified Rankin Scale
STA: superficial temporal artery

## 1 Introduction

Moyamoya angiopathy (MMA) is a cerebrovascular disease in which the main arteries of the anterior cerebral circulation are gradually narrowed until occlusive with characteristic abnormal vascular networks [1,2]. Currently, the most effective treatment for MMA is to improve the symptoms of intracranial ischemia through cerebral blood flow (CBF) reconstruction [3,4]. The main surgical methods are divided into three types: direct, indirect, and combined cerebral reconstruction. Its main significance lies in surgical intervention to supplement the cerebral blood supply to the intracranial ischemic area. Improving the supply of CBF will have an impact on cerebral hemodynamic balance [3,5,6]. Although this kind of new postoperative complication caused by changes in intracranial hemodynamics occurs in a small number of patients, the consequences are often catastrophic and will have a significant impact on the clinical prognosis of patients [7,8,9].

Arterial spin labeling (ASL) is an emerging magnetic resonance technology. The procedure provides better convenience, higher reliability, higher repeatability, and lower risk of cerebral hemodynamic monitoring, as compared with other methods [10,11,12]. At present, many studies on the ASL technology of MMA mainly focus on the detection of CBF in children with MMA and the advantages compared with other technologies [13,14,15,16]. The aim of this study is to use ASL technology to monitor perioperative cerebral hemodynamic changes in patients with MMA and to explore the application of ASL technology in combined flow reconstruction surgery and its potential application value in the long-term follow-up of patients.

## 2 Materials and methods

### 2.1 Patient selection

A retrospective cohort study was conducted to select patients with MMA from the Neurosurgery Moyamoya Disease Diagnosis and Treatment Center from September 2019 to August 2021. The random number method was used for selection. All the patients were diagnosed using digital subtraction angiography (DSA). The diagnostic criteria were as follows: (1) bilateral ICA end and/or ACA and/or MCA initial segment stenosis or occlusion and (2) an abnormal vascular network at the skull base appearing in the arterial phase [17]. Angiography results were evaluated by two neurointerventional physicians.

The inclusion criteria were as follows: (1) patient with stable condition before the operation and no new cerebral hemorrhage, cerebral infarction, frequent transient ischemic attacks (>2 times per week), and other related neurological symptoms; (2) patient underwent the first CBF reconstruction surgery (“superficial temporal artery (STA)–middle cerebral artery (MCA) bypass and encephalo–duro–myo–synangiosis”) in our hospital, performed by the same surgeon; (3) patient was treated before surgery, and at 1 week and 6 months after surgery; (4) the MRI machine in our hospital performed ASL perfusion imaging monitoring; and (5) patient agreed to perform ASL assessment and signed an informed consent form.

**Figure 1:**
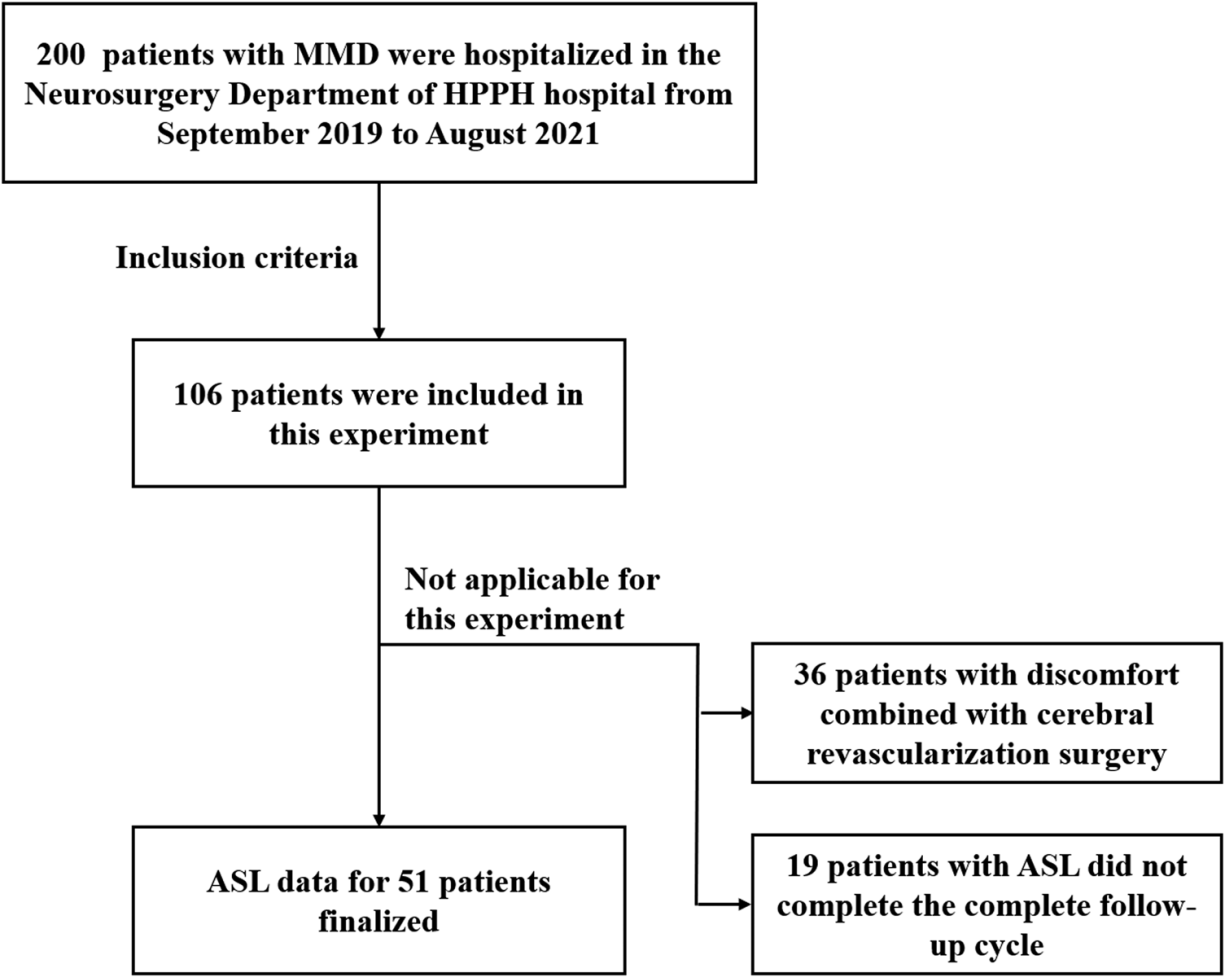
Patient inclusion flowchart.

The exclusion criteria were as follows: (1) patients with Graves’ disease, antiphospholipid antibody syndrome, systemic vasculitis, Sjogren’s syndrome, systemic atherosclerosis, and other clear comorbidities; (2) DSA examination of patients with MMA accompanied by other brain vascular diseases, such as aneurysms and cerebrovascular malformations; (3) inability to perform ASL imaging due to personal constitutions, such as mental abnormalities, claustrophobia, cognitive impairment; (4) the quality of ASL imaging of the patient was poor or does not perform standard imaging; (5) the patient’s clinical data were missing or incomplete; and (6) The patient did not develop serious postoperative complications, such as massive cerebral infarction, cerebral hemorrhage, or grand mal seizure (Figure 1).

### 2.2 Surgical procedure

After general anesthesia, according to the individual conditions of the patient, blood pressure and partial pressure of carbon dioxide were controlled [18,19,20]. The extended pterional approach was used; the temporal muscle and STA were carefully separated, the bone flap was removed, and the dura mater was carefully cut to expose the brain surface. A microscope (ZEISS) was used to perform a fluorescence contrast test to understand the blood flow of the distal middle cerebral artery on the brain surface and select the appropriate blood vessels. The recipient blood vessel and STA branch were wrapped and soaked with papaverine solution to prevent vasospasm. Subsequently, a temporary aneurysm clip was used to clamp the blood supply on both sides of the recipient blood vessel and proximal end of the STA branch. The frontal parietal surface was examined at the distal end of the body vessel and the STA branch. After bypass was completed, intraoperative angiography was performed to check the patency and flow of the reconstructed vessel. Finally, the separated and intact temporal muscles were directly applied to the parietal and frontal brain surfaces, and the dura mater was turned over and applied to the brain surface to complete the combined cerebral revascularization operation [21,22].

### 2.3 ASL image acquisition

The ASL technology used a Philips Ingenia 3.0T magnetic resonance scanner (Philips, the Netherlands). The patient was placed in the supine position, while the head was properly fixed, and a 32-channel head special coil was used. The scanning baseline was the front and back joint connection, and the scanning sequence was axis T1WI, axis T2WI, axis T2 FLAIR, and pseudo-continuous arterial spin labeling (pCASL). The scanning parameters of the pCASL sequence were as follows: repetition time = 4,364 ms; echo time = 12 ms; layer thickness, 6 mm; field of view, 240 mm × 240 mm; matrix, 64 × 60; delay time after marking, 2,000 ms; number of excitations, 1; and scanning time, 306 s. The CereFlow ASL image software (Animage Technology, China) was used for post-image processing. The processing flow was as follows: (1) the ASL original map calculated CBF through a single-chamber model; (2) the cerebral perfusion parameter map was standardized to the MNI brain template space [23]; (3) the brain atlas of the standard grading system was covered based on the Alberta Stroke Program Early CT Score (ASPECT) of Alberta stroke procedures, covering ten regional divisions of the MCA blood supply area at two levels: the nucleus level (thalamus and striatum plane) divided into seven areas: medial prearterial cortex (M1), middle cerebral artery extrainsular cortical area (M2), posterior middle cerebral cortex (M3), insula, lentiform nucleus, caudate nucleus, capsula interna, and the level above the nucleus (add cmMo at the nucleus level). There were three areas of the middle cerebral artery cortex above M1 (M4), the middle cerebral artery cortex above M2 (M5), and the middle cerebral artery cortex above M3 (M6) [24]. (4) After covering the brain atlas, the mean value of each area in each brain perfusion parameter map was analyzed. The absolute quantitative CBF value of the aspect area on the operation side was automatically obtained.

### 2.4 Revascularization classification

To assess the revascularization effect, Matsushima grading [25] was used to classify and compare the patients into two groups. First, the hypovascular group had the following characteristics: (1) no obvious change in the diameter of the bypass vessel or obvious intracranial compensation; (2) the bypass vessel was thickened or a small amount of compensation was performed on the brain, and (3) the compensation of the MCA area on the operation side was less than 1/3. Second, the hypervascular group included the following: (1) the bypass vessel compensated for the cranium, and for the MCA area between 1/3 and 2/3 on the operation side; and (2) the bypass vessel compensated for more than 2/3 of the MCA area on the operation side. According to the Alberta stroke grade and CBF value, the stroke score was given (Table 1).

**Table 1:** Alberta stroke grading criteria

Area	Score	Area	Score
Insula	1	M5	1
M1	1	M6	1
M2	1	Caudate nucleus	1
M3	1	Capsule inner	1
M4	1	Lenticular nucleus	1

Albert stroke score was 10 points, each area lower than normal blood flow (CBF <30 mL/100 g/min) [26] was defined as the perfusion defect area, and the corresponding brain area score was reduced by 1 point, and the final score was calculated.

### 2.5 Statistical analysis

All data were entered and analyzed using SPSS (version 26.0, IBM, USA). Corresponding statistical analysis of the general clinical data and CBF data was performed. Measurement data (such as ASL monitoring time and age) and data conforming to the normal distribution are expressed as mean value ± standard deviation (\bar{x}\pm s] ), and paired *t*-tests were used to analyze the data. The measurement data that did not conform to the normal distribution adopted the median/quartile (M/P25, P75) and were analyzed using the Wilcoxon non-parametric test. The enumeration data (sex, clinical manifestations, etc.) were analyzed using the chi-square test and Fisher’s exact test. Grade data (such as Suzuki staging) were tested using a rank-sum test. Statistical significance was set at *P* ≤ 0.05.


**Ethical approval:** The research related to human use has been complied with all the relevant national regulations, institutional policies and in accordance with the tenets of the Helsinki Declaration, and has been approved by the authors’ institutional review board or equivalent committee. This study was reviewed and approved by the Ethics Committee of Henan Provincial People's Hosptial (2020183).
**Informed consent:** Informed consent has been obtained from all individuals included in this study and the clinical data were kept strictly confidential.

## 3 Results

### 3.1 Demographics

Based on the inclusion and exclusion criteria, 51 patients with 90 hemispheres were included in this study. Table 1 summarizes the baseline participants and clinical characteristics of MMA. The mean age on admission was 41.68 ± 11.38 years and men (60.78%) constituted the majority of the cohort. The clinical presentation showed onset symptoms of ischemia in 37 (72.54%) patients and intracranial hemorrhage in 14 (27.46%) patients. The ASL monitoring time was recorded on three postoperative periods: first, 4.39 ± 1.49 days; second, 6.26 ± 0.67 days; and third, 181.65 ± 10.93 days (Table 2).

**Table 2:** Baseline characteristics of the included patients

Characteristics	All cases
No. of patients	51
No. of hemispheres^a^	90
Age (years)^b^	41.68 ± 11.38
Sex^a^	
Male	31 (60.78%)
Female	20 (39.22%)
Premorbid history^a^	
Hypertension	32 (62.74%)
Diabetes	16 (31.37%)
CHD	9 (17.64%)
Smoker	29 (56.86%)
Hemisphere involved^a^	
Bilateral	39 (76.47%)
Right	7 (13.72%)
Left	5 (9.81%)
Primary type^a^	
Ischemic	37 (72.54%)
Hemorrhagic	14 (27.46%)
Admission mRS^a^	
0–2	45 (88.23%)
3–6	6 (11.77%)
Surgical side^a^	
Right	46 (51.11%)
Left	44 (48.89%)
Suzuki stage^a^	
2	5 (5.56%)
3	45 (50.00%)
4	37 (41.11%)
5	3 (3.33%）
ASL time	
First^b^	4.39 ± 1.49
Second^b^	6.26 ± 0.67
Third^b^	181.65 ± 10.93

ASL: arterial spin labeling, CHD: chronic heart disease. ^a^Percentage (%). ^b^Mean value ± SD.

### 3.2 ASL perfusion data

The CBF values of each region of the cerebral hemisphere before the operation were: insula, 29.06 ± 9.85; M1, 26.06 ± 11.67; M2, 27.74 ± 10.24; M3, 27.29 ± 9.70; M4, 24.98 ± 12.18; M5, 25.71 ± 9.97; M6, 27.25 ± 11.35; caudate nucleus, 21.67 ± 9.34; inner capsule, 20.85 ± 6.81; and lenticular nucleus, 29.22 ± 12.09.

The CBF values of each region of the cerebral hemisphere after 1 week of operation on the surgical side were as follows: insula, 47.74 ± 22.50; M1, 32.78 ± 14.02; M2, 36.14 ± 12.81; M3, 34.35 ± 14.53; M4, 33.72 ± 15.34; M5, 33.50 ± 14.62; M6, 35.32 ± 16.28; caudate nucleus, 21.85 ± 6.76; inner capsule, 24.63 ± 7.39; and lenticular nucleus, 33.14 ± 11.29.

The CBF values of each region of the cerebral hemisphere on the surgical side at 6 months after the operation were: insula, 34.52 ± 12.19; M1, 29.51 ± 13.47; M2, 33.78 ± 15.31; M3, 33.15 ± 11.09; M4, 29.14 ± 13.70; M5, 29.07 ± 12.71; M6, 31.38 ± 11.24; caudate nucleus, 20.78 ± 7.90; inner capsule, 19.10 ± 5.39; and lenticular nucleus, 24.84 ± 5.74.

**Figure 2:**
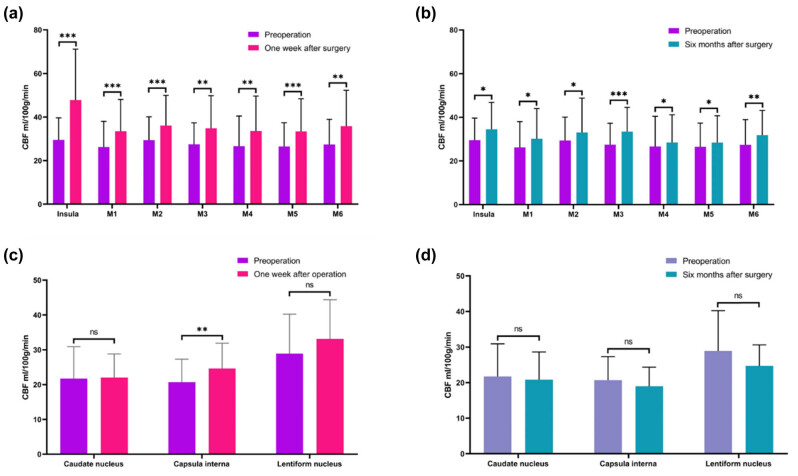
Quantitative CBF values in each area of enrolled patients were evaluated under ASPECT partitioning criteria. (a and b) The CBF values of each cerebral cortex region were statistically different (preoperation vs 1 week after surgery, preoperation vs 6 months after surgery). (c and d) The CBF values of each subcortical region were statistically different (preoperation vs 1 week after surgery, preoperation vs 6 months after surgery). Paired *t* test was used for comparison between the two groups, and *P* ≤ 0.05 was considered to be statistically significant. ns: *P* > 0.05, *: *P* ≤ 0.05, **: *P* < 0.01, ***: *P* < 0.001.

After applying the paired *t*-test to the three sets of data, the preoperative and 1week postoperative CBF value comparison showed that there were statistical differences in eight regions: the insula, M1, M2, M3, M4, M5, M6, and inner capsule. There was no statistical difference between the caudate nucleus and lenticular nucleus. Comparison of the preoperative and 6 months postoperative CBF values showed statistical differences in seven regions: the insula, M1, M2, M3, M4, M5, and M6. There was no statistical difference in the caudate nucleus, inner capsule, and lenticular nucleus (Figure 2).

### 3.3 Relationship between ASPECT score and the effect of postoperative cerebrovascular reconstruction

**Figure 3:**
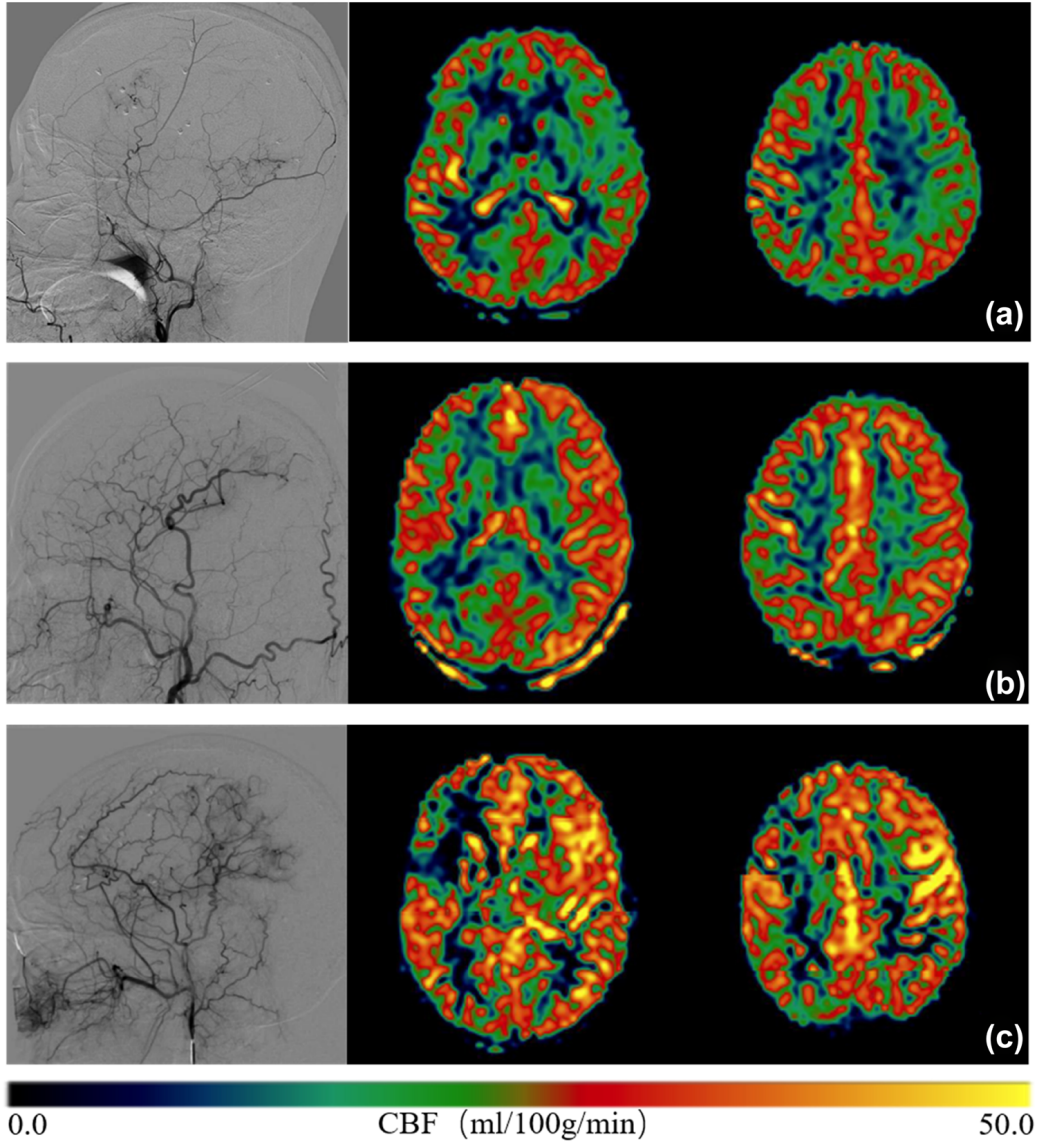
The postoperative CBF of the patients reach the normal level under different vascular conditions. (a–c) Relationship between ASL images and postoperative neovascularization network density in three typical patients. The Alberta score of the three patients decreased gradually before surgery, and the neovascularization network density increased gradually after surgery.

According to the Matsushima classification, the 90 hemispheres were divided into 2 groups: 69 in the hypovascular group and 21 in the hypervascular group. Statistical differences in variables between the groups were compared. There was no statistically significant difference in the general clinical symptoms between the two groups (*P* > 0.05). Among the three Alberta scores, the preoperative Alberta score differed between the two groups (*P* < 0.05). At the same time, we compared the changes in the mRS scores between the two groups before and after surgery. There was a significant difference between the two groups before surgery (*P* ≤ 0.05) (Table 3, Figure 3).

**Table 3:** Comparing differences between hypovascular and hypervascular groups

Category	Total (*n* = 90)	Good (*n* = 69)	Poor (*n* = 21)	Inspection value	*P* value
**Alberta score**					
Preoperative^a^	2.70 ± 3.35	1.36 ± 2.30	4.78 ± 3.76	2.714	0.013
One week after operation^a^	4.96 ± 2.65	5.44 ± 2.87	4.86 ± 1.46	0.650	0.523
Six months after operation^a^	5.31 ± 2.13	5.36 ± 1.94	5.22 ± 3.56	−0.118	0.907
**mRS score**					
Preoperative^a^	3.13 ± 0.90	3.42 ± 0.75	1.55 ± 0.71	6.678	<0.001
Six months after operation^a^	1.51 ± 0.69	1.55 ± 0.71	1.38 ± 0.59	0.986	0.280

^a^Mann–Whitney U test was used for statistical methods, and *P* ≤ 0.05 was considered to be statistically significant.

## 4 Discussion

From a global perspective, MMA is a relatively rare cerebrovascular disease. However, in China, with the continuous advancement of medical technology and the increasing importance of medical care among people, the detection rate of MMA has shown an upward trend annually. At present, CBF reconstruction surgery is still the most effective treatment for MMA patients, which can reduce the incidence of stroke and improve long-term prognosis [17,27]. However, reconstruction surgery, especially direct and combined reconstruction, can effectively change the CBF supply immediately after the operation, but the operation will disrupt the balance of intracranial hemodynamics. To know more and accurate assessment of the preoperative and postoperative intracranial cerebral hemodynamic status, timely discovery of the risk of postoperative local cerebral hypoperfusion or high perfusion, and evaluation of long-term CBF have become important issues that urgently need to be solved. The emergence of ASL technology has brought us new evaluation methods.

CBF is very important for cerebral function, CBF reconstruction is an effective surgical method for the treatment of MMA. Combined reconstruction not only applies direct anastomosis between the STA and MCA on the operation side but also involves attaching the dura mater and temporal muscle to the brain surface, so that varieties of blood supply channels are used to supply blood to the brain. However, STA–MCA direct bypass has a greater impact on the balance of cerebral hemodynamics, and early postoperative patients are prone to hyperperfusion syndrome [28]. Hayashi et al. [29] found that CBF changes within postoperative 2 weeks in patients with MMA. Therefore, CBF changes within 2 weeks was classified as early postoperative moyamoya disease, and after 2 weeks was classified as the late postoperative period. In our current study, our first postoperative review time was 6.26 ± 0.67 days, which was in the early postoperative range of MMA. Significant statistical differences were found in eight areas: the insula, M1, M2, M3, M4, M5, M6, and the capsula interna. CBF in all areas of the MCA increased, especially in the insula, from 29.06 ± 9.85 before the operation to 47.74 ± 22.50, and the cerebral cortex of the MCA area on the operation side showed a significant improvement in perfusion. Using ASL technology, the improved CBF can be effectively and accurately measured after direct STA–MCA bypass. Sugino et al. [30] found that in two patients with MMA who had transient deterioration of neurological function due to hyperperfusion syndrome after direct CBF reconstruction, ASL showed a significant increase in CBF in the focal part of the operative side, suggesting the importance of ASL in monitoring postoperative hyperperfusion syndrome. At present, there is no definite conclusion regarding the range of CBF changes in patients with MMA after surgery for hyperperfusion syndrome. The application of ASL to evaluate CBF changes in patients with hyperperfusion syndrome preoperatively and 1 week after surgery may have a certain predictive value for the prevention of postoperative hyperperfusion syndrome.

To check whether blood flow changed long after revascularization, we followed about 6 months by using ASL technology. Cho et al. [31] found in the CBF study by ASPECT that after cerebral revascularization, the compensatory abnormal blood vessel network of the skull base gradually begins to subside after at least 6 months, and the new compensatory blood vessels tend to be stable and play a major role in the blood supply. The growth range of the new blood vessels can be as long as 54 months. In this study, all patients underwent DSA and ASL reexamination 6 months after the operation. In the comparison of ASL monitoring preoperatively and 6 months after the operation, we found that the change in CBF in the cortical area of the cerebral MCA was still statistically significant, whereas the changes in the caudate nucleus, capsula interna, and lenticular nucleus were not significant. The CBF in all MCA cortical areas reached the normal level, while the caudate nucleus, capsula interna, and lenticular nucleus were still lower than the normal level at 6 months post operation. Our study findings are similar to those of Cho et al., but ASL technique does not require radionuclides and is noninvasive.

Six months after surgery, the new compensatory vessels played a stable compensatory role on the surgical side, and the CBF in the cerebral cortex returned to normal. However, owing to the gradual disappearance of the abnormal skull base vascular network, the CBF in the deep area of the MCA still fails to reach the normal level. Based on the aforementioned research findings, it has been observed that there is no significant improvement in deep blood flow among patients with smoke disease post-surgery. As a result, there is a need for a novel surgical approach to enhance deep cerebral blood flow, particularly for those who have experienced hemorrhage in the lenticular and caudate regions.

Since MMA is a vascular occlusive disease, we were very concerned about vessel growth after revascularization. During disease evolution, an intracranial compensatory vascular network is essential to supplement the ischemic area. Therefore, in this study, we uniformly calibrated the PLD time to 2 s to obtain the CBF value and calculated the Alberta stroke score before and 6 months after the operation. Simultaneously, the reconstructed vessels were graded using DSA. A correlation was found between Alberta and Matsushima ratings. A low preoperative Alberta score may have a beneficial effect on the growth of reconstructed vessels postoperatively. Previous studies have found that for stroke patients, a good preoperative collateral circulation may lead to significant postoperative cerebrovascular reconstruction [32]. But from our results, there was a negative correlation between blood vessel growth and preoperative CBF.

Meanwhile, in the present study, no patient exhibited deterioration in the mRS score following bypass. A low preoperative Alberta score indicates that the affected side of the brain has a large range of ischemic states with a poor collateral circulation compensation state. The effect of postoperative vascular reconstruction is relatively good. This phenomenon may indicate that patients with severe preoperative ischemia have a strong demand for extracranial compensatory vessels (Figure 3).

In this study, the preoperative, postoperative, and long-term prognosis of CBF in patients with MMA were monitored through the application of ASL technology, the results proved that ASL technique is an effective method for CBF measurement. This trial may provide a more convenient, fast, and efficient imaging evaluation methods for MMA patients. Moreover, this study provides evidence of a significant association between the quantity of neovessels in the surgical region and preoperative CBF values among patients diagnosed with smoke disease. Those who exhibit greater preoperative ischemia are likely to derive more substantial benefits from revascularization surgery.

### 4.1 Limitations

The aim of this study was to provide data that ASL technology can contribute as a practical and significant method for patients with MMA and can play an auxiliary role in monitoring postoperative complications. However, owing to the small sample size and short follow-up time, the results may be biased. It is still necessary to expand the sample size and conduct more long-term follow-up monitoring of patients to continue to explore the application value of ASL technology in MMA.

## 5 Conclusion

In this study, the comparison of ASL in the MCA cortical region after 1 week and 6 months from operation showed that there was no significant difference in other regions, except the insula and M4. CBF increased significantly at 1 week after operation. At 6 months postoperatively, CBF was established and maintained with long-term stability and effectiveness. The Alberta score is correlated with the area of postoperative cerebral vascular reconstruction, and the effect of postoperative cerebral revascularization is better in patients with a lower preoperative Alberta score.
